# Primary intraosseous osteolytic meningioma without an evidence of soft tissue invasion

**DOI:** 10.1002/ccr3.3694

**Published:** 2020-12-18

**Authors:** Ali Adnan Dolachee, Samer S. Hoz, Ghazwan A. Lafta

**Affiliations:** ^1^ Department of Neurosurgery College of Medicine AL‐Qadisiyah University Al Diwaniyah Iraq; ^2^ Neurosurgery Department Neurosurgery Teaching Hospital Baghdad Iraq; ^3^ Department of Surgery Faculty of Medicine University of Al‐Ameed Karbala Iraq

**Keywords:** extradural meningioma, osteolytic meningioma, primary intraosseous meningioma, total resection

## Abstract

Primary intraosseous osteolytic meningiomas are not common, and total surgical removal is the best treatment option if the location allows that; however, long‐term follow‐up is recommended since it may recur.

## Graphical abstract

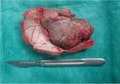

## 1 INTRODUCTION

In this article, we present a case of intraosseous osteolytic meningioma and describe the steps of management. A 45‐year‐old obese woman presented with headache only for six months, and skull lesion was diagnosed. Total surgical removal was done, and the histopathology result revealed meningioma. Primary intraosseous meningiomas are usually benign, and their growth correlates with their histological grade and can show malignant changes more than its intracranial equivalent. Total surgical resection is the treatment of choice with safe margins. A long‐term follow‐up period is recommended even if total resection is carried out.

Meningiomas are the most frequent intracranial benign neoplasia; they arise from arachnoid granulations and represent approximately from 14% to 20% of intracranial tumors.^1^ In 1904, Winkler described for the first time a meningioma with extradural location that later they denominated ectopic or extradural meningiomas.^2^ This tumor subtype represents approximately 1% to 2% of extradural meningiomas (EM). This variant emerges outside from nervous system, generally in intracranial bones. Primary intraosseous meningiomas (PIM) are infrequent tumors that represent 14%‐67% of EM. However, they are rarely presented as a skull osteolytic lesion, whereby the diagnosis is usually performed wrongly and they are confused with primary osseous tumor. ^3^ PIM has been defined as a lesion that does not involve the dura mater. ^4^

In this article, we present a case of intraosseous osteolytic meningioma and describe the steps of management.

## 2 CASE PRESENTATION

**Figure 1:**
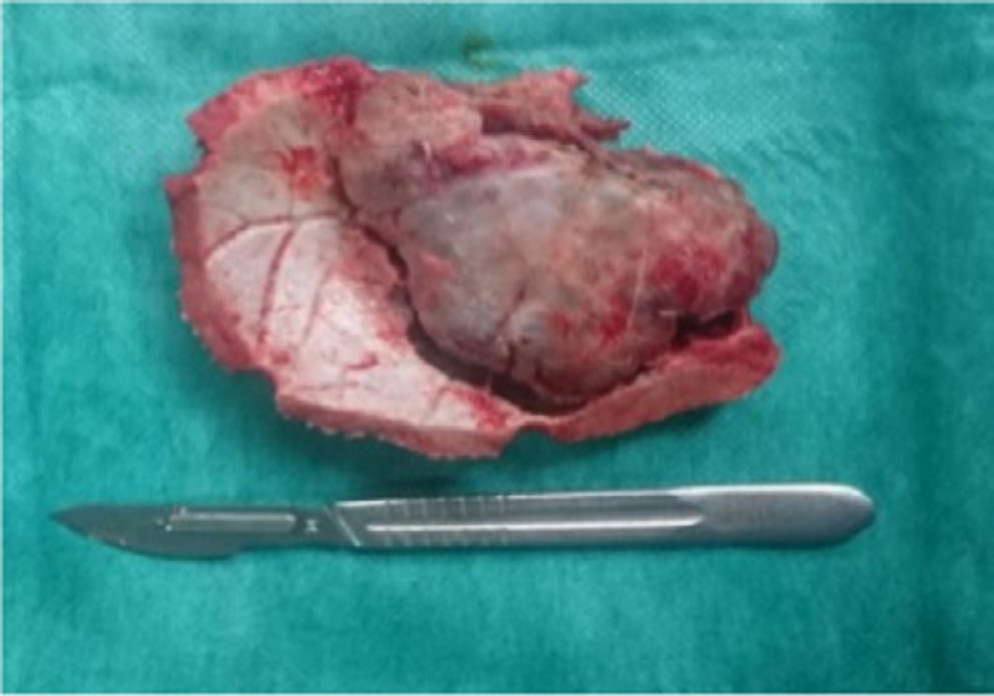
Intraoperative picture showing total removal of tumor with its bony attachment

A 45‐year‐old obese woman with history of diabetes mellitus and hypertension presented with headache for six months of duration. Neurological examination was normal, and there was no scalp mass or deformity. Computed tomography scan (CT scan) revealed hypodense (osteolytic) lesion occupying the right temporoparietal region with destruction of inner and outer table, differential diagnosis was suspected and includes fibrous dysplasia, aneurysmal bone cyst, and primary intraosseous meningioma. She underwent magnetic resonance imaging (MRI) that revealed hypointense in T1 and hyperintense in T2 with homogenous contrast enhancement lesion in the right temporoparietal region with mild compression of the right ventricle, which most likely represent a meningioma. Surgical resection was planned; a large question mark skin incision was made, after periosteal dissection and reflection of temporalis muscle; en bloc resection of tumor with safe margins was done; dura was intact; we control the feeders of the tumor, which were mainly from middle meningeal artery branches, wound closed in layers; and cranioplasty was postponed for another time to shorten the operation time due to patient medical condition (Figure 1). Postoperative brain CT scan showed complete resection, and the histopathology result confirmed the diagnosis of meningioma.

## 3 DISCUSSION

This neoplasm has no gender predilection, and it has two peaks of incidence in the second decade and between the fifth and seventh decades with a median age of 40‐49 years,^5^ and this is consistent with our patient who presented with 45 years old. The most common locations are the convexity and skull base.^6^ The most frequent places of PIM are frontotemporal orbits, anterior cranial fossa, paranasal sinuses, nasal cavity, neck, and petrous region of the temporal; our location occupied the skull convexity. There are several classifications for primary EM. However, the following has been postulated: type 1 (purely extracalvarial), type 2 (purely calvarial), and type 3 (calvarial lesion that extends beyond the cranial vault), and each type presents subgroups for skull base and convexity.^4^ According to the above, intraosseous meningiomas can be considered primary EM of type 2 or type 3,^6^ and we can consider our patient as type 2. Intraosseous meningiomas are divided into osteoblastic, osteolytic, and mixed according to the imaging characteristics,^4^ and in our case, the lesion was osteolytic. There are several types of intraosseous meningiomas according to their microscopic appearance, of which we can find transitional, microcystic, cardioid, psamomatous, fibroblastic, atypical, and meningothelial, the latter being the most common histological type.^6^ The clinical manifestations of PIM depend to a large extent on the size, location of the lesion, and the mass effect.^3^, ^6^ The majority of intraosseous meningiomas in the base of the skull and orbit are usually asymptomatic, but may present pain, proptosis, and neurological symptoms.^3^ Neoplasms at the base of the skull can cause cranial nerve deficit, ophthalmoplegia, visual field defect, and /or signs and symptoms generated by the mass effect; fortunately, our patient suffered from only headache with normal neurological examination all over the time before surgery.^7^ Generally, these neoplasms are benign and slow‐growing, although there are atypical meningiomas with malignant characteristics. Malignant presentation is more frequently compared with intradural meningiomas, with osteolytic tumors being more prone to have atypical characteristics with respect to osteoblastic tumors, despite being the latter more frequent.^6^, ^7^ The features of malignancy include increased mitotic activity, increased cellularity, papillary characteristics, necrosis foci, giant cells, and involvement of the dura mater and overlying soft tissues. For this reason, the osteolytic variant of meningiomas has an evolution characterized by greater aggressiveness. Differential diagnoses of intraosseous mass include multiple myeloma, giant cell tumor, chondroma, chondrosarcoma, eosinophilic granuloma, metastatic cancer, dermoid tumor, aneurysmal bone cyst, hemangioma, and fibrous dysplasia.^6^ To differentiate osteolytic intraosseous meningiomas from the above conditions, imaging techniques such as MRI should be used. Also, MRI with gadolinium enhancement allows excluding lesions such as hemangiomas and eosinophilic granuloma, which do not enhance after contrast medium administration. However, the rapid histological diagnosis of these neoplasms is necessary due to their high capacity to present malignancy compared with other meningiomas.^8^ Osteolytic lesions are characterized by presenting as a hypodensity on CT, unlike osteoblastic lesions where the characteristic finding is hyperdensity.^4^, ^6^ In CT, a narrowing expansion and interruption of the internal and external cortical layers of the skull can be observed when it is an osteolytic meningioma. CT with bone windows is the most used technique since it identifies the expansion of the neoplasm and the osteolytic lesions.^3^ MRI is hypointense in T1 images, unlike in T2 where these tumors are hyperintense, with a homogeneous uptake after administering a contrast. In addition, the "dural tail" that can be observed in intradural meningiomas is not appreciated.^2^, ^6^ Bone scintigraphy with technetium‐99m diphosphonate has been very useful to determine the results of adjuvant therapy in patients with malignant tumors that were not resected.^3^, ^7^ The treatment of choice in most cases of primary intraosseous osteolytic meningioma (PIOM) is complete surgical resection, However, this procedure cannot be achieved in all cases of PIOM.^9^ Simultaneous to resection, cranial reconstruction can be performed when necessary.^10^ When total tumor resection is not possible, as in lesions located at the base of the skull, the paranasal sinuses, or the orbit, the objective of the procedure will be to reduce compression to important neuronal structures^4^, ^7^; in our case, a total surgical removal was performed as the location of the tumor was totally exposed and no vital structures were involved; hence, total resection was feasible. Regarding tumor recurrence, long‐term monitoring of the patient after the treatment is essential. It has been reported that benign ones have their recurrence up to 10 years after the operation.^6^

## 4 CONCLUSION

Primary intraosseous meningiomas are usually benign, and their growth correlates with their histological grade and can show malignant changes more than its intracranial equivalent. Total surgical resection is the treatment of choice with safe margins. If there is doubt about complete resection, the lesion should be followed with appropriate imaging studies. A long‐term follow‐up period is recommended even if total resection is carried out.

## CONFLICT OF INTEREST

The authors declare no conflict of interest; the manuscript is not published elsewhere.

## AUTHOR CONTRIBUTIONS

AD and SH: conceived and designed the study. GL: involved in overall supervision of the paper. All authors: read and approved the final manuscript.
